# Supplementary material for: Comprehensive Profiling of Primary and Metastatic ccRCC Reveals a High Homology of the Metastases to a Subregion of the Primary Tumour
Source: Cancers (Basel). 2019 Jun 12;11(6):812. doi: 10.3390/cancers11060812 (PMC6628027; doi:10.3390/cancers11060812)
Supplement: Supplementary file 1 [file cancers-11-00812-s001.pdf]

Supplementary materials

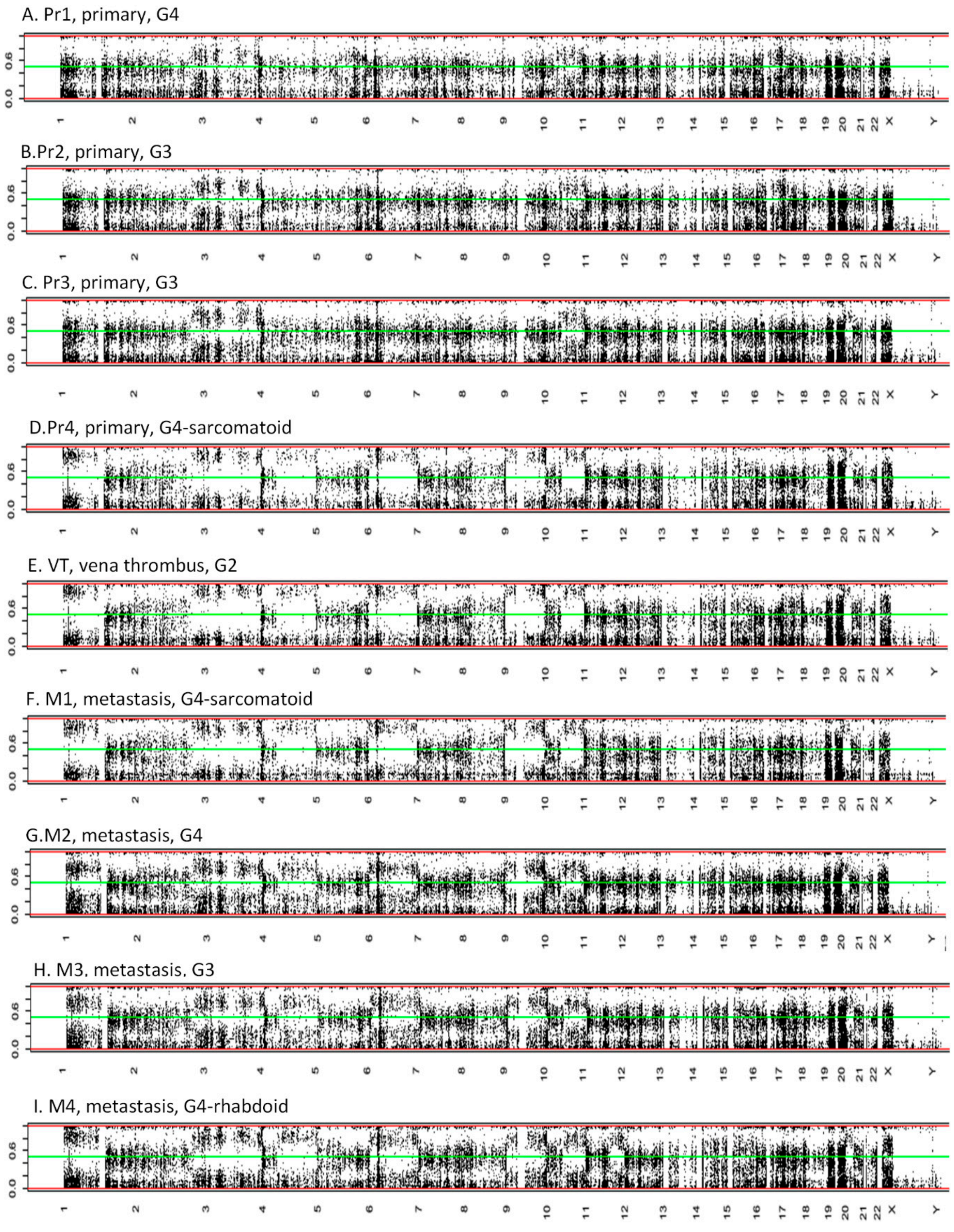

Figure S1. B-allele frequency (BAF) plots of all tumour samples of patient RC1. X-axes represent the genomic position starting from 1pter until Xqter. Y-axes indicate B-allele frequency. Regions with a B-allele frequencies around 0.5 have an even number of copies, including a normal diploid state.

Table S1. Minor allele frequency median of normal and all tumour samples of Patient RC1.

Chromosome	Normal	Pr1	Pr3	Pr2	Pr4	VT	M1	M2	M3	M4
1p	46	43	41	44	14	12	15	26	24	18
3	46	31	25	27	14	11	14	26	25	17
4p	46	45	41	45	45	44	45	43	44	42
4q	46	44	40	43	33	23	22	33	31	23
6	45	43	43	43	15	11	14	27	26	22
13	46	42	44	43	42	40	42	44	43	38
8	46	44	44	44	37	36	36	40	39	42
14	46	43	44	44	38	36	36	41	40	35
15	45	43	43	44	36	35	36	41	39	33

ArrayCGH based copy number changes: yellow-normal; red-loss; green-gain.

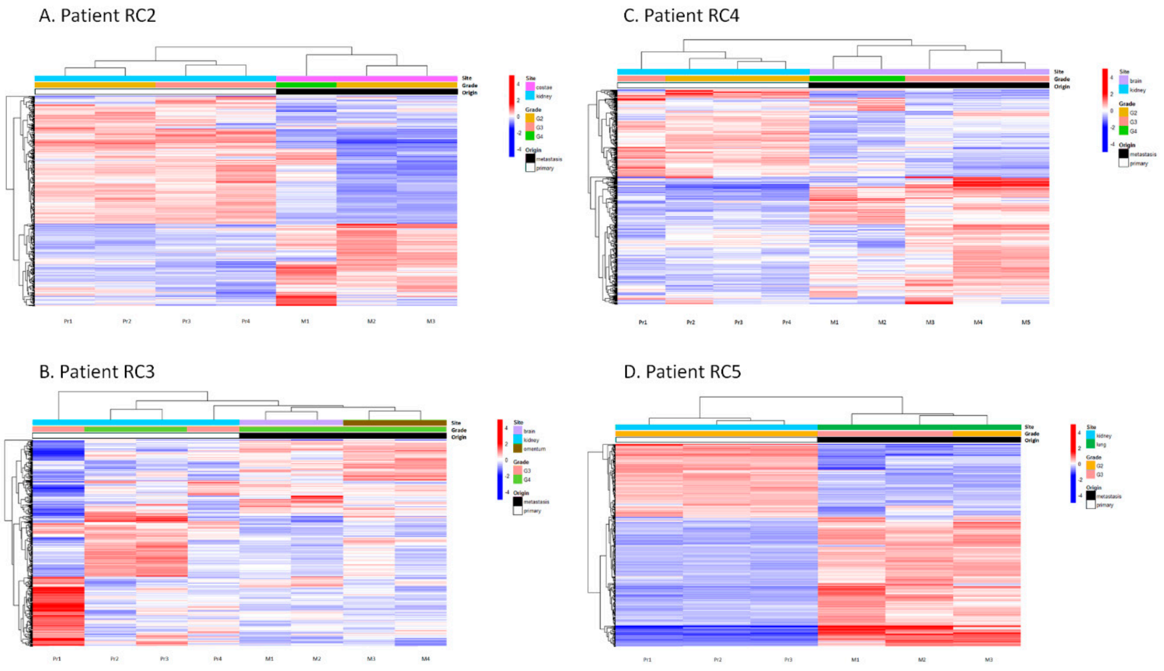

Figure S2. Unsupervised hierarchical clustering based on the 500 genes with the highest variance in gene expression across samples from Patient RC2 (A), RC3 (B), RC4 (C) and RC5 (D). X-axis represents the tumour samples. For each gene, the mean value across the samples was defined. Expression levels higher than the mean value are shown in red. Expression levels lower than the mean value are indicated in blue. The site of origin and tumour grade are indicated by the colours at the top of the Figure. The overall similarity between tumour samples is depicted by the dendrogram at the top of the figure, which is based on the measurement of Euclidian distance between tumour samples in expressing genes.

Table S2. Whole exome sequencing quality report of Patient RC1.

Samples	Raw Reads	Reads Aligned	Unique Reads Aligned	Average Sequencing Depth on Target	10× Coverage
Normal	49×10^6	99%	89%	68	89%
VT	48×10^6	99%	88%	53	87%
Pr1	56×10^6	99%	84%	53	87%
Pr2	55×10^6	99%	88%	63	89%
Pr3	67 × 106	99%	84%	68	93%
Pr4	46×10^6	99%	88%	50	83%
M1	50×10^6	99%	86%	51	87%
M2	67×10^6	99%	85%	82	94%
M3	59×10^6	99%	83%	65	94%
M4	68×10^6	99%	81%	64	95%

Table S3. Validation of somatic mutations by targeted sequencing.

Position	Variant	Gene	MRF of each position detected by targeted sequencing									Number of total cases (major, minor, absent)	Number of cases with depth ≥10	Number of concordant cases
**2:222347390**	**T>G**	***EPHA4***	**0.43**	**0.29**	0.62	0.50	0.36	1.00	0.88	0.50	0.67	9	5	5
3:9776257	A>C	*BRPF1*	0.34	0.33	0.51	0.72	0.63	0.67	0.47	0.46	0.57	9	9	9
3:10183872	G>A	*VHL*	0.43	0.50	0.45	0.50	0.71	0.50	0.45	0.58	0.39	9	9	9
3:52668692	G>C	*PBRM1*	0.75	0.54	0.29	0.67	0.57	0.75	0.43	0.54	0.55	9	5	5
7:44152235	G>A	*AEBP1*	0.39	0.41	0.38	0.40	0.47	0.32	0.32	0.29	0.17	9	9	9
7:141464317	T>A	*TAS2R3*	0.50	0.57	0.14	0.67	0.14	0.54	0.45	0.15	0.44	9	4	4
11:67888338	A>C	*CHKA*	0.32	0.22	0.43	0.43	0.42	0.27	0.31	0.24	0.37	9	9	9
12:56397956	T>A	*SUOX*	0.16	0.33	0.19	0.27	0.29	0.26	0.23	0.20	0.30	9	9	9
16:89929993	T>A	*SPIRE2*	0.45	0.39	0.25	0.33	0.43	0.39	0.36	0.21	0.19	9	9	9
20:44472264	T>A	*ACOT8*	0.33	0.34	0.41	0.39	0.73	0.42	0.35	0.38	0.39	9	9	9
7:36489359	C>CT	*ANLN*	0.00	0.67	0.20	0.00	0.42	0.44	0.29	0.22	0.25	9	4	4
14:21542614	A>G	*ARHGEF40*	0.32	0.50	0.44	0.27	0.38	0.13	0.65	0.00	0.40	9	8	7
19:1510186	GC>G	*ADAMTSL5*	0.22	0.25	0.38	0.27	0.23	0.31	0.00	0.20	0.27	9	9	8
3:10420932	C>T	*ATP2B2*	0.00	0.04	0.42	0.82	0.86	0.79	0.51	0.60	0.19	9	9	9
12:108959131	C>T	*ISCU*	0.00	0.07	0.32	0.50	0.39	0.40	0.46	0.29	0.40	9	8	7
10:17107512	G>T	*CUBN*	0.00	0.00	0.00	0.17	0.38	0.26	0.36	0.20	0.33	9	8	8
2:66664909	G>T	*MEIS1*	0.46	0.39	0.42	0.26	0.31	0.24	0.21	0.19	0.24	9	9	9
11:14666132	T>C	*PDE3B*	0.27	0.27	0.00	0.00	0.00	0.07	0.00	0.00	0.00	9	9	9
18:8826172	C>G	*SOGA2*	0.23	0.33	0.00	0.00	0.00	0.00	0.00	0.00	0.07	9	9	9
1:27092746	A>G	*ARID1A*	0.25	0.50	0.38	0.00	0.00	0.00	0.00	0.00	0.00	9	5	5
6:161771211	G>T	*PARK2*	0.00	0.01	0.01	0.35	0.00	0.01	0.00	0.02	0.02	9	9	9
1:180165714	G>T	*QSOX1*	0.33	0.00	0.00	0.00	0.00	0.00	0.00	0.00	0.00	9	9	9
15:58001316	C>T	*GCOM1*	0.00	0.00	0.00	0.00	0.00	0.00	0.02	0.10	0.00	9	8	7
5:139228177	CGCCG>C	*NRG2*	0.19	0.00	0.00	0.00	0.00	0.00	0.00	0.00	0.00	9	9	9
6:56371328	CCATG>C	*DST*	0.50	0.25	0.75	NA	0.00	0.00	0.00	0.00	0.00	9	0	0
1:6534175	G>C	*PLEKHG5*	0.00	0.00	0.00	0.00	0.00	0.00	0.00	0.00	0.18	9	9	9
17:7577121	G>A	*TP53*	0.12	0.00	0.00	0.00	0.00	0.00	0.00	0.00	0.00	9	8	8
			SUM									243	207	203
			Concordant cases											0.98

MRF: mutant read frequency. Grey highlight: read depth in targeted sequencing <10. Yellow highlight: non-concordant case between WES and targeted sequencing.

Table S4. RNA sequencing quality report of five ccRCC patients.

Patient	Sample code	Total reads	Aligned reads	500 bases reads
RC1	RC1.33 (Pr3)	10268960	7443978	2447269
	RC1.30 (Pr1)	10497617	6149293	2111509
	RC1.31 (Pr2)	11364514	7526812	2531602
	RC1.32 (Pr4)	10967443	2866431	460411
	RC1.29 (VT)	12859540	4220238	1935315
	RC1.35 (M1)	11535288	7471822	2866891
	RC1.39 (M2)	18746572	9106495	2701171
	RC1.36 (M3)	13665301	8031682	2528924
	RC1.37 (M4)	17653037	9002634	3273343
RC2	RC2.41 (Pr3)	14709306	4474411	1189480
	RC2.42 (Pr2)	18933981	4178741	1154163
	RC2.43 (Pr1)	15756495	2523947	597345
	RC2.48 (Pr4)	13887927	4982475	1486999
	RC2.45 (M3)	12669026	5678896	1833549
	RC2.44 (M4)	14415909	7462411	2448312
	RC2.46 (M5)	14850348	5974482	1788465
	RC2.2 (M1)	11173392	4861329	1102690
	RC2.3 (M2)	15352885	4473227	674382
RC3	RC3.5 (Pr1)	12343993	8634239	2632904
	RC3.6 (Pr2)	14253020	3588264	677181
	RC3.7 (Pr4)	13802033	8632195	2965246
	RC3.8 (Pr3)	10705178	2679318	460537
	RC3.12 (M3)	17853948	4813354	1063875
	RC3.13 (M4)	22218649	6321194	1695892
	RC3.10 (M1)	14724481	8666084	3115233
	RC3.11 (M2)	13737557	10835843	3987932
RC4	RC4.16 (Pr1)	19847051	10011136	3527256
	RC4.17 (Pr2)	21068870	7717536	2838737
	RC4.14 (Pr3)	18815382	8602789	2907266
	RC4.15 (Pr4)	19729956	7218359	2137288
	RC4.18 (M2)	10421183	8934146	3153718
	RC4.19 (M1)	15990148	9201591	3062552
	RC4.21 (M3)	13676291	6156225	2296343
RC5	RC5.23 (Pr2)	13084300	7948791	2776428
	RC5.24 (Pr3)	11249225	7660983	2586524
	RC5.26 (Pr1)	16320929	9235257	3480370
	RC5.22 (M1)	14230045	8869762	2802021
	RC5.47 (M2)	14078902	10370479	3851834
	RC5.25 (M3)	19022834	7194171	2438752

Table S5. Differentially expressed genes primary vs. metastasis groups in five ccRCC patients.

	Gene Symbol	Mean Coverage (*)	Fold Changes (=Mets/Primary) (*)	p-value	pAdj (*)	DAVID Functional Analysis
ENSG00000133392	*MYH11*	160	0.19	1.98E-15	1.61E-11	
ENSG00000188060	*RAB42*	55	4.92	1.04E-13	4.22E-10	
ENSG00000157766	*ACAN*	82	0.26	5.61E-13	1.52E-09	ECM
ENSG00000181577	*C6orf223*	73	0.29	2.17E-12	3.53E-09	
ENSG00000147642	*SYBU*	110	3.73	4.70E-12	5.45E-09	
ENSG00000170370	*EMX2*	82	0.15	5.44E-12	5.52E-09	
ENSG00000125740	*FOSB*	299	4.47	5.65E-11	2.87E-08	
ENSG00000053702	*NRIP2*	52	0.25	2.22E-10	8.56E-08	
ENSG00000152377	*SPOCK1*	202	4.06	1.33E-08	2.62E-06	
ENSG00000079689	*SCGN*	736	0.13	1.45E-08	2.68E-06	
ENSG00000047457	*CP*	1934	3.10	2.16E-08	3.82E-06	BM
ENSG00000172348	*RCAN2*	142	0.26	2.90E-08	4.70E-06	
ENSG00000136352	*NKX2-1*	89	23.92	5.79E-08	8.10E-06	
ENSG00000073792	*IGF2BP2*	107	4.17	6.36E-08	8.61E-06	
ENSG00000075891	*PAX2*	305	0.23	9.40E-08	1.17E-05	
ENSG00000175793	*SFN*	61	4.08	1.12E-07	1.35E-05	
ENSG00000113083	*LOX*	572	3.16	1.28E-07	1.52E-05	ECM
ENSG00000060718	*COL11A1*	135	6.15	1.35E-07	1.57E-05	ECM
ENSG00000196549	*MME*	141	3.29	2.62E-07	2.60E-05	
ENSG00000188373	*C10orf99*	63	15.78	6.37E-07	5.12E-05	
ENSG00000146555	*SDK1*	61	3.10	7.75E-07	6.00E-05	
ENSG00000145824	*CXCL14*	707	0.26	1.46E-06	9.98E-05	
ENSG00000166292	*TMEM100*	75	4.06	1.94E-06	1.26E-04	
ENSG00000103569	*AQP9*	123	3.46	3.09E-06	1.74E-04	
ENSG00000011465	*DCN*	687	3.41	5.23E-06	2.70E-04	ECM
ENSG00000171759	*PAH*	77	0.14	6.71E-06	3.22E-04	
ENSG00000256870	*SLC5A8*	128	0.21	6.99E-06	3.34E-04	

	Gene Symbol	Mean Coverage (*)	Fold Changes (=Mets/Primary) (*)	p-value	pAdj (*)	DAVID Functional Analysis
ENSG00000178828	*RNF186*	201	0.33	8.11E-06	3.78E-04	
ENSG00000135525	*MAP7*	101	0.32	8.87E-06	4.03E-04	
ENSG00000107317	*PTGDS*	65	3.73	1.15E-05	4.84E-04	
ENSG00000124253	*PCK1*	388	0.27	1.39E-05	5.70E-04	
ENSG00000083067	*TRPM3*	71	0.22	2.39E-05	8.55E-04	
ENSG00000140284	*SLC27A2*	52	0.33	3.22E-05	1.06E-03	
ENSG00000196569	*LAMA2*	52	3.48	3.95E-05	1.24E-03	ECM
ENSG00000124107	*SLPI*	51	5.86	4.28E-05	1.33E-03	
ENSG00000174564	*IL20RB*	94	4.00	4.50E-05	1.36E-03	
ENSG00000157005	*SST*	73	0.08	5.04E-05	1.46E-03	
ENSG00000128591	*FLNC*	72	3.18	5.29E-05	1.50E-03	
ENSG00000196136	*SERPINA3*	338	4.29	5.27E-05	1.50E-03	BM
ENSG00000160282	*FTCD*	100	0.26	8.25E-05	2.10E-03	
ENSG00000171560	*FGA*	503	4.79	8.33E-05	2.10E-03	ECM & BM
ENSG00000173432	*SAA1*	246	3.89	1.01E-04	2.43E-03	
ENSG00000189058	*APOD*	56	5.28	1.04E-04	2.47E-03	
ENSG00000133661	*SFTPD*	74	5.28	1.78E-04	3.73E-03	
ENSG00000148942	*SLC5A12*	90	0.18	1.79E-04	3.74E-03	
ENSG00000171564	*FGB*	988	5.78	1.80E-04	3.74E-03	ECM & BM
ENSG00000152268	*SPON1*	177	0.27	2.44E-04	4.68E-03	
ENSG00000171885	*AQP4*	57	3.97	2.52E-04	4.77E-03	
ENSG00000081479	*LRP2*	633	0.28	2.77E-04	5.12E-03	
ENSG00000130234	*ACE2*	112	0.20	4.08E-04	6.61E-03	
ENSG00000168484	*SFTPC*	638	8.40	4.53E-04	7.19E-03	
ENSG00000173698	*GPR64*	160	3.89	4.62E-04	7.29E-03	
ENSG00000144908	*ALDH1L1*	66	0.27	4.84E-04	7.53E-03	
ENSG00000013588	*GPRC5A*	72	3.48	5.20E-04	7.99E-03	

	Gene Symbol	Mean Coverage (*)	Fold Changes (=Mets/Primary) (*)	p-value	pAdj (*)	DAVID Functional Analysis
ENSG00000144035	*NAT8*	444	0.24	5.42E-04	8.26E-03	
ENSG00000160161	*CILP2*	60	3.84	5.81E-04	8.67E-03	
ENSG00000163631	*ALB*	102	0.22	6.18E-04	9.05E-03	BM

Abbreviation: ECM, extracellular matrix organization; BM, blood microparticle. (*) criteria for selected genes: absolute mean coverage >50, fold changes <3, and adjective p value after multiple testing correction/Benjamin-Hochberg algorithm (pAdj) <0.01.

Supplementary Methods

Patient RC-1 Medical History and Sample Origin

A male patient with diagnosis of clear cell renal cell carcinoma (ccRCC) pT3bNoM1 underwent surgery for a primary tumour resection in the right kidney. Four samples were taken from the primary tumour; a tumour region with tumour grade 4 (Pr1), tumour grade 3 (Pr2), tumour grade 4-sarcomatoid differentiation (Pr4), and tumour grade 3 (Pr3). Also a tumour thrombus reaching inferior vena cava was resected (sample VT with tumour grade 2). In the same year the patient underwent wedge excision for the metastatic lesions in the right and the left lungs. Three samples were taken from this procedure; a metastasis in the lingula of the left lung with tumour grade 4-sarcomatoid differentiation (M1), a metastasis in the dorsal apex, lower lobe of the left lung with tumour grade 4 (M2), a metastasis in the lateral basal, the under lobe of the right lung with tumour grade 3 (M3). A year later a recurrent metastatic lesion was found in the left lung. The patient received conventional immunotherapy of PEG-interferon with a good response. Two years later, the patient underwent resection of the whole upper lobe of the left lung (M4 with tumour grade 4-rhabdoid).

DNA and RNA Isolation of FFPE Blocks

The FFPE blocks were serially cut in 10 µm slides, in which the first and the last slides (3 µm in thickness) were stained with H&E and used as reference in identifying tumour regions with a different WHO/ISUP grade. The odd cut slides were processed for DNA isolation and the even slides were processed for RNA isolation. The isolation of DNA was performed following the Adaptive Focused Acoustics™-based DNA extraction of FFPE/ truXTRAC™ FFPE DNA kit protocol (Covaris, Woburn, MA, USA) and RNA isolation was performed following the Adaptive Focused Acoustics™-based RNA extraction of FFPE/ truXTRAC™ FFPE microTUBE RNA kit protocol (Covaris, Woburn, MA, USA).

Variant Filtering of Whole Exome Sequencing (WES) Data

Called variants were annotated using snpeff/snpsift 3.5 [1], with the Ensembl release 75 gene annotations [2], 1000 genome phase 1, dbNSFP2.7 [3], and ExAC 0.3 databases [4]. The annotated variants were filtered using the following exclusion criteria: mutant allele frequency >2% in the 1000 genome project phase 1 or >0.01% in ExAC database, the possibility of error >1/100 in calling (QUAL < 20), low quality by depth (QD < 2 and QD/AF < 8.0), strand bias (FS > 60 for SNVs and >200 for Indels), present in tandem repeat units (RPA > 8), present in normal sample (personal variants), putative non harmful variant e.g., synonymous variants, and variants located in non-coding regions.

Correction Based on Tumour Cell Purity and Somatic Mutation Identification

The mutant read frequency (MRF) of each variant detected by WES was corrected for the normal cell admixture. The approximate percentage of normal cells in each sample was calculated based on the read counts for personal variants on the short arm of chromosome 3. As arrayCGH data indicated

loss of 3p in all samples except M4, all tumour cells contribute only one copy of the personal variant, whereas all normal cells contribute one copy of both alleles for a certain personal variant. M4 appears to have two identical copies of chromosome 3. Thus the imbalance between both alleles can be used to estimate the percentage of tumour cells. The MRF of all variants in each sample was recalculated based on the percentage of total reads belonging to the tumour cells.

For each patient, the mutations present in each tumour area were classified into major clonal, minor clonal, absent, or inconclusive [5]. If the total number of mutant reads was ≥5 and the MRF ≥ 25%, the mutation was considered to be a major clone. If the total number of mutant reads was ≥5 and the MRF < 25%, or the total number of mutant reads was ≥3 and the total read count was ≥ 10, the mutation was defined as a minor clone. If the total number of mutant reads was < 3 and the total read count was ≥ 10, the mutation was defined as absent. Every mutation with total read count < 10 was considered as inconclusive. Matched normal kidney samples were included to remove personal variants in WES and TS data. The Integrative Genomic Viewer (IGV) was used to confirm the authenticity of the identified somatic mutations [6].

Ploidy Estimations Based on Median BAFs of the Germline Variants

We used the SNV allele counts for the germline variants to get more information on the ploidy state of each tumour sample. The germline variants were selected based on having a B- allele frequency (BAF) 0.4–0.5 in the normal sample. We calculated the BAF of all variants in tumour samples and determined the median BAF for a selection of chromosome, or chromosomal segments (supplementary table 1). The median BAF for the germline variants in the normal sample is 0.46 and is representative of an even number of chromosomal copies. If in a tumour sample the median BAF for any segment is close to this value, this indicates that also in the tumour cells the absolute copy number for this genomic segment is even. If this occurs for the lowest ploidy level in an array CGH plot this means that this level represents a copy number of 2. In contrast, if the median BAF in a tumour sample is very different from 0.46, this indicates either an odd number of copies or an isodisomic situation.

Somatic Mutation Validation by Targeted Sequencing for

To validate the somatic mutations previously detected by WES in Patient RC-1, a targeted sequencing assay based on Single Primer Enrichment Technology (SPET) has been designed using the Ovation™ Custom Target Enrichment System (NuGEN, San Carlos, CA, USA). Landing probes were designed close to, and on both sides of the selected mutations (see supplementary Table 3). The library preparation was done according to the FFPE-specific protocol from the manufacturer (NuGEN, San Carlos, CA). Single-end sequencing of enriched libraries was performed on the Illumina NextSeq 550 System (Illumina, San Diego, CA, USA).

Generation of Phylogenetic Trees

Unsupervised hierarchical clustering was done in R 3.4.0 with the ape 5.0 package using the binary distance matrix based on the presence or absence of amplification/deletion in each chromosome arm generated by arrayCGH.

RNAseq Reads Processing and Gene Expression Analysis

In the processing of RNAseq reads, FASTQ files were polyA and polyG trimmed. Then, the reads were aligned to the reference genome (grch37 1000 genomes reference build with decoy sequences from the GATK bundle ([7,8] with ensembl version 75 transcript annotation [9] using hisat [10]. General read operations were performed using SAMtools [11]. The gene-level quantification was performed using Htseq-count [12]. The analysis of gene expression was done per patient in R 3.4.0 using DESeq2 package, which is based on negative binomial generalized linear models [13]. Only genes with a mean read count ≥50 across all samples of a patient were used for further analysis.

Unsupervised gene clustering, based on the 500 most highly variable expressed genes across all samples in each patient, was done to compare the gene expression profiles in each patient. Regularized-logarithm transformation (rlog) was used to transform the count data to the log2 scale which minimizes differences between samples with small counts of genes, and which normalizes according to library size. The heat map was constructed based on the amount of deviation of each gene in a specific sample from the mean expression of that particular gene in all samples. The colour ranges for gene expression was adjusted with the rlog -2 to 2 as the core and rlog -6 to 6 as the extension.

Supervised gene clustering analysis was used to identify genes differentially expressed between primary tumours and metastases from all five patients, in which P-values were adjusted for multiple testing correction (Benjamin-Hochberg algorithm). A Padj-value ≤0.01 was considered statistically significant.

References:

1. Cingolani, P.; Platts, A.; Wang le, L.; Coon, M.; Nguyen, T.; Wang, L.; Land, S. J.; Lu, X.; Ruden, D. M. A program for annotating and predicting the effects of single nucleotide polymorphisms, SnpEff: SNPs in the genome of Drosophila melanogaster strain w1118; iso-2; iso-3. *Fly (Austin)* **2012**, *6*, 80–92.
2. Consortium, G. P.; Abecasis, G. R.; Auton, A.; Brooks, L. D.; DePristo, M. A.; Durbin, R. M.; Handsaker, R. E.; Kang, H. M.; Marth, G. T.; McVean, G. A. An integrated map of genetic variation from 1092 human genomes. *Nature* **2012**, *491*, 56–65.
3. Liu, X.; Jian, X.; Boerwinkle, E. dbNSFP v2.0: a database of human non-synonymous SNVs and their functional predictions and annotations. *Hum. Mutat* **2013**, *34*, 2393–2402.
4. Lek, M.; Karczewski, K. J.; Minikel, E. V.; Samocha, K. E.; Banks, E.; Fennell, T.; O'Donnell-Luria, A. H.; Ware, J. S.; Hill, A. J.; Cummings, B. B.; et al. Exome Aggregation, C., Analysis of protein-coding genetic variation in 60,706 humans. *Nature* **2016**, *536*, 285–91.
5. Saber, A.; Hiltermann, T. J. N.; Kok, K.; Terpstra, M. M.; de Lange, K.; Timens, W.; Groen, H. J. M.; van den Berg, A. Mutation patterns in small cell and non-small cell lung cancer patients suggest a different level of heterogeneity between primary and metastatic tumors. *Carcinogenesis* **2017**, *38*, 144–151.
6. Robinson, J. T.; Thorvaldsdottir, H.; Winckler, W.; Guttman, M.; Lander, E. S.; Getz, G.; Mesirov, J. P. Integrative genomics viewer. *Nat. Biotechnol.* **2011**, *29*, 24–26.
7. DePristo, M. A.; Banks, E.; Poplin, R.; Garimella, K. V.; Maguire, J. R.; Hartl, C.; Philippakis, A. A.; del Angel, G.; Rivas, M. A.; Hanna, M.; et al. A framework for variation discovery and genotyping using next-generation DNA sequencing data. *Nat. Genet.* **2011**, *43*, 491–498.
8. Van der Auwera, G. A.; Carneiro, M. O.; Hartl, C.; Poplin, R.; Del Angel, G.; Levy-Moonshine, A.; Jordan, T.; Shakir, K.; Roazen, D.; Thibault, J.; et al. From FastQ data to high confidence variant calls: the Genome Analysis Toolkit best practices pipeline. *Curr. Protoc. Bioinformatics* **2013**, *43*, doi:10.1002/0471250953.bi1110s43.
9. Flicek, P.; Amode, M. R.; Barrell, D.; Beal, K.; Billis, K.; Brent, S.; Carvalho-Silva, D.; Clapham, P.; Coates, G.; Fitzgerald, S.; et al. Ensembl 2014. *Nucleic Acids Res.* **2014**, *42*, D749–755.
10. Kim, D.; Langmead, B.; Salzberg, S. L. HISAT: a fast spliced aligner with low memory requirements. *Nat. Methods* **2015**, *12*, 357–360.
11. Li, H.; Handsaker, B.; Wysoker, A.; Fennell, T.; Ruan, J.; Homer, N.; Marth, G.; Abecasis, G.; Durbin, R.; 1000 Genome Project Data Processing Subgroup. The Sequence Alignment/Map format and SAMtools. *Bioinformatics* **2009**, *25*, 2078–2079.
12. Anders, S.; Pyl, P. T.; Huber, W. HTSeq--a Python framework to work with high-throughput sequencing data. *Bioinformatics* **2015**, *31*, 166–169.
13. Love, M. I.; Huber, W.; Anders, S. Moderated estimation of fold change and dispersion for RNA-seq data with DESeq2. *Genome Biol.* **2014**, *15*, 550.
